# Fertility and parturition parameters in beef type heifers

**DOI:** 10.1007/s11250-025-04505-5

**Published:** 2025-06-09

**Authors:** Ayşe Uysal, Vefa Tohumcu, Soner Uysal, Ali Sarı, Oğuz Zenginoglu, Mehmet Cengiz, Armağan Hayırlı

**Affiliations:** 1 Department of Animal Science, Faculty of Veterinary Medicine, Ataturk University, Erzurum, 25240 Turkey; 2 Department of Obstetrics and Gynecology, Faculty of Veterinary Medicine, Ataturk University, Erzurum, 25240 Turkey; 3 Department of Animal Nutrition and Nutritional Disorders, Faculty of Veterinary Medicine, Ataturk University, Erzurum, 25240 Turkey; 4 Doğan Beef Production Farm, Gümüşhane, 29600 Turkey; 5 Department of Obstetrics and Gynecology, Faculty of Veterinary Medicine, Sıtkı Koçman University, Muğla, 48000 Turkey

**Keywords:** Beef type, Fertility, Heifer, Parturition

## Abstract

This study compared fertility and parturition parameters in Limousin (L) and Charolais (C) heifers to identify the breed most suitable for beef production. A total of 234 L and 76 C Hungarian-origin heifers, aged 9–11 months, were managed under standardized housing and feeding conditions at Dogan Farm. Parturitions were categorized based on the time of day (day: 07:00–19:00; night) and season (cold, cool, hot). Statistical analyses were conducted using ANOVA and Chi-square tests Results showed that C heifers required significantly more inseminations to conceive (C: 3.39 ± 0.22, L: 2.32 ± 0.10, *P* < 0.0001), indicating lower reproductive efficiency. Additionally, C calves were significantly heavier at birth than L calves (C: 46.1 ± 0.8 kg, L: 40.2 ± 0.3 kg, *P* < 0.0001), with male calves being generally heavier than females (M: 43.7 ± 0.5 kg, F: 39.5 ± 0.3 kg, *P* < 0.0001). The incidence of dystocia was higher in C heifers (63.16%) compared to L heifers (41.88%), while L heifers experienced greater parturition ease (58.12% vs. 36.84%; X^2^ = 10.4244, *P* < 0.0054).

## Introduction

Meat and milk derived from livestock represent a substantial proportion of the total protein intake for humans, playing a vital role in global food security (Thornton 2010). To address the increasing global demand for meat production, strategies such as genetic selection, nutritional optimization, and effective reproductive management are imperative for sustainably enhancing cattle meat production (Terry et al. 2020). The sustainability of cattle farming heavily relies on healthy parturitions and successful reproduction, which are pivotal for maintaining productivity and profitability in this sector (Moorey and Biase 2020). Reproductive efficiency, in particular, is critical for cattle producers, as animals without reproductive problems significantly contribute to operational success and economic viability. This highlights fertility as a key trait in the cattle industry (Stegemiller et al. 2021).

Fertility is a multifaceted process influenced by factors such as the insemination index (number of services per conception) (Smulski et al. 2020), age at conception, age at first parturition, and gestation period. These factors work together as elements that determine a cow's reproductive potential (Hu et al. 2023). Achieving one calf per year is considered the optimal parturition interval for most farming systems; however, dairy farms may extend this interval depending on milk yield priorities (Burgers et al. 2021). Conversely, for beef breeds where calf production is the primary focus, shorter parturition intervals are desired to maximize productivity (Bene et al. 2022). Among beef cattle, C and L are prominent breeds recognized for their superior meat quality and production efficiency (Kayar and Inal 2022). Understanding the fertility and parturition parameters of these two significant breeds within beef cattle operations is essential for optimizing farm management strategies. The reproductive performance of C and L heifers reflects the interaction of genetic traits, environmental conditions, and management practices employed during fattening (Kotnik et al. 2009).

Parturition represents a critical phase in cattle farming, with numerous parameters serving as indicators of cattle health and survival outcomes. Key parturition parameters such as dystocia incidence, GP, calf weight, parturition type, and retention rate provide invaluable insights into reproductive health and management efficiency. Routine monitoring of these parameters enables beef cattle operations to refine parturition management strategies and improve overall herd performance. Additionally, these metrics are vital for evaluating the effects of parturition processes on calf health, offering practical implications for both breeders and veterinarians (Silva et al. 2020a, b; Mahnani et al. 2021). This study aims to compare the fertility and parturition parameters of C and L heifers to determine which breed demonstrates greater advantages for beef production systems. Through this analysis, the research seeks to provide evidence-based recommendations for optimizing cattle farming practices.

## Materials and methods

### Animals, housing, located and feeding

The study material comprised L (*n* = 248) and C (*n* = 81) heifers aged 9–11 months, sourced from Hungary, all of which had undergone the required legal, transportation, and welfare procedures prior to sale. After completing a quarantine period, the heifers were housed at Dogan Beef Farm (25°22′35″S, 52°07′45″E) in pens of 40 animals each, grouped based on age and size. During the experimental period, the average temperature was 6.5 °C, with an annual rainfall of 578 mm. The animals were fed a total mixed ration (TMR) formulated to meet their nutritional requirements, distributed in two daily feedings. Housing, management, and reproductive conditions were standardized across all experimental groups. Heifers were inseminated upon reaching a body weight of 390–400 kg at approximately 15 months of age. Before parturition, 14 L (5.65%) and 5 C (6.17%) heifers were culled for various reasons. The study focused on the remaining heifers (L, *n* = 234 and C, *n* = 76), which were confirmed to be free of any residual metabolic or gynecological disorders.

### Fertility and parturition parameters

The reproductive and parturition performance of heifers was evaluated across two primary categories. Fertility parameters were assessed under the subheadings Age at conception, Natural/Artificial insemination index (number of inseminations required per conception), and Age at first parturition, while Parturition parameters were evaluated under the subheadings Calf weight, Parturition ease, Retained placenta rate, Day and night parturitions, Complications, and Seasonal classification.

### Groups, experimental protocols and seasons

L and C heifers were grouped to compare fertility and parturition parameters. To achieve this, a double-prostaglandin F2α synchronization protocol was applied, and artificial insemination was performed either during synchronization-induced estrus or spontaneous estrus. Parturitions occurring between 07:00 and 19:00 were classified as day parturitions, while those outside this timeframe were categorized as night parturitions. To evaluate the influence of season on reproductive performance, inseminations and parturitions were grouped into three distinct seasonal periods: Cool periods included April, May, September, and October; Cold periods comprised November through March; and Hot periods encompassed June, July, and August. Placental expulsion within the first 24 h post-parturition was classified as non-retained, whereas expulsions occurring after this period were categorized as retained. Calving complications were classified into three groups—Uterine Prolapse, Trauma, and None—to assess the effects of factors such as breed, season, parturition ease, and calf sex.

### Statistical analysis

All statistical analyses were performed using the Statistical Analysis System (SAS, version 9.0, Cary, NC, USA). Before performing the one-way ANOVA, the data were tested for normality and homogeneity. Data normality was assessed using the Shapiro–Wilk test. For normally distributed data, one-way analysis of variance (ANOVA) was used to compare continuous variables between breeds. When significant differences were detected, post-hoc tests were applied to determine pairwise differences. Relationships between categorical variables were evaluated using the Chi-square (χ^2^) test. Statistical significance was set at *P* < 0.05. Results are reported as mean ± standard deviation (SD) for continuous variables and as percentages (%) for categorical variables.

## Results

In the study comparing the fertility and parturition parameters of C and L heifers, the insemination index of C heifers was significantly higher than that of L heifers. Conversely, L heifers achieved a higher pregnancy rate with fewer inseminations.

No significant differences were found between C and L heifers in terms of age at conception (AC) (C: 538 ± 10 days, L: 544 ± 6 days), age at first parturition (AFP) (C: 822 ± 10 days, L: 829 ± 6 days), or gestation length (GL) (C: 284 ± 0.5 days, L: 285 ± 0.6 days) (Table 1). C heifers required significantly more inseminations to conceive compared to L heifers (C = 3.39 ± 0.22 vs. L = 2.32 ± 0.10, *P* < 0.0001; Table 1). The birth weight (BW) of C calves was significantly higher than that of L calves (C = 46.1 ± 0.8 kg, L = 40.2 ± 0.3 kg, *P* < 0.0001; Table 1). Male calves were heavier than female calves (Male = 43.7 ± 0.5 kg, Female = 39.5 ± 0.3 kg, *P* < 0.0001; Table 1). No significant interactions between breed and calf sex were observed for GL, BW, or AFP (Table 1).

**Table 1:** The effect of breed and calf sex on pregnancy parameters in beef heifers

Factor		Parameter^*^				
Breeds	Calf sex	AC (Day)	N/A II	GL (Day)	AFP (Day)	CBW (kg)
Charolais		538 ± 10	3.39 ± 0.22	284 ± 0.5	822 ± 10	46.1 ± 0.8
Limousin		544 ± 6	2.32 ± 0.10	285 ± 0.6	829 ± 6	40.2 ± 0.3
	Female	-	-	284 ± 0.6	833 ± 8	39.5 ± 0.3
	Male	-	-	285 ± 0.7	821 ± 6	43.7 ± 0.5
Charolais	Female	-	-	284 ± 0.8	837 ± 16	43.7 ± 1.0
	Male	-	-	285 ± 0.7	811 ± 12	47.9 ± 1.1
Limousin	Female	-	-	284 ± 0.8	832 ± 8	38.3 ± 0.2
	Male	-	-	286 ± 0.9	825 ± 8	42.1 ± 0.5
ANOVA		*P* <				
Breeds		0.6105	< 0.0001	0.6834	0.6598	< 0.0001
Calf sex		-	-	0.2516	0.1504	< 0.0001
Breeds * Calf sex		-	-	0.6463	0.4214	0.7614

^*^Data are LS means ± SE, AC: Age at conception, N/A II: Natural/Artificial insemination index, *GL* Gestation length, *AFP* Age at first parturition, *CBW* Calf birth weight

The relationship between breed, conception season, and parturition season was analyzed using the Chi-square test, as presented in Table 2. No statistically significant association was found between breed and conception season (*P* = 0.3393). C heifers had similar proportions of conceptions during the cold (42.11%), cool (43.42%), and hot seasons (14.47%), whereas L heifers showed a higher proportion of conceptions during the cool season (47.44%) compared to the cold (33.33%) and hot seasons (19.23%). In contrast, a marginal association was observed between breed and parturition season (*P* = 0.0868). C heifers displayed a more uniform distribution of parturitions across seasons, with 31.58% occurring during the cold season, 26.32% during the cool season, and 42.11% during the hot season. L heifers had the highest percentage of parturitions during the cold season (45.73%), followed by the hot (31.62%) and cool seasons (22.65%) (Table 2).

**Table 2:** Association between breed, conception, and parturition season

		Season			^♣^Statistics
Relationship	^*^ Breeds	Cold	Cool	Hot	
Breed X conception season	Charolais	32 (%42.11)	33 (%43.42)	11 (%14.47)	X^2^ = 2.1617 *P* < 0.3393
	Limousin	78 (%33.33)	111 (%47.44)	45 (19.23)	
Breed X parturition season	Charolais	24 (%31.58)	20 (%26.32)	32 (%42.11)	X^2^ = 4.8878 *P* < 0.0868
	Limousin	107 (%45.73)	53 (%22.65)	74 (%31.62)	

^♣^Chi-square test was used to evaluate the relationship between breed and conception/parturition seasons, ^*^Charolais: *n* = 76, Limousin: *n* = 234

A significant difference in dystocia rates was observed between breeds, with C heifers exhibiting a higher dystocia rate (63.16%) compared to L heifers (41.88%) (Table 3). Similarly, parturition ease was greater in L heifers (58.12%) compared to C heifers (36.84%) (X^2^ = 10.4244, *P* < 0.0054; Table 3). No significant differences were observed between breeds regarding complications. When examining natural versus synchronized insemination protocols, C heifers conceived more frequently following natural estrus (56.58%), whereas L heifers showed a higher conception rate following synchronization protocols (76.50%, *P* < 0.0001; X^2^ = 29.0282; Table 3).

**Table 3:** The relationship between breed and parturition ease, complications, protocol for insemination, parturition time, calf sex, and retained placenta rate

^*^ Breeds	Relationship			^♣^Statistics
	**Parturition ease**			
	Easy	Difficult	Very difficult	
Charolais	28 (%36.84)	47 (%61.84)	1 (%1.32)	X^2^ = 10.4244 *P* < 0.0054
Limousin	136 (%58.12)	96 (%41.03)	2 (%0.85)	
	**Complications**			
	Uterine prolapse	Trauma	No	
Charolais	3 (%3.95)	0 (%0)	73 (%96.05)	X^2^ = 1.9872 *P* < 0.3702
Limousin	9 (%3.85)	6 (%2.56)	219 (%93.59)	
	**Protocol for insemination**			
	Natural estrus	Synchronization		
Charolais	43 (%56.58	33 (%43.42)		X^2^ = 29.0282 *P* < 0.0001
Limousin	55 (%23.50)	179 (%76.50)		
	**Parturition time**			
	Night parturitions	Day parturitions		
Charolais	34 (%44.74)	42 (%55.26)		X^2^ = 0.0937 *P* < 0.7596
Limousin	100 (%42.74)	134 (%57.26)		
	**Calf sex**			
	Female	Male		
Charolais	33 (%43.42)	43 (%56.58)		X^2^ = 0.7534 *P* < 0.3854
Limousin	115 (%49.15)	119 (%50.85)		
	**Retanined placenta rate**			
	Expelled	Not expelled		
Charolais	73 (%96.05)	3 (%3.95)		X^2^ = 0.4586 *P* < 0.4983
Limousin	220 (%94.02)	14 (%5.98)		

^♣^Chi-square test was used to analyze the relationships presented in the table, ^*^Charolais (*n* = 76) and Limousin (*n* = 234) were the breeds included in the study

No significant differences were found between breeds regarding parturition time, calf sex, or retained placenta rate (Table 3). The pregnancy rate within the first three services was higher in L heifers (82.48%) compared to C heifers (59.22%) (X^2^ = 27.5870, *P* < 0.0003; Table 4). The number of inseminations varied by season, with more inseminations performed during extreme hot and cold seasons (X^2^ = 27.1338, *P* < 0.0185; Table 4). Parturition was easier for female calves compared to male calves (X^2^ = 25.8450, *P* < 0.0001; Table 5). However, no relationship was found between calf sex and complications, parturition time, or retained placenta rate (Table 5).

**Table 4:** The relationship between natural/artificial number of insemination and breed and season

	Relationship				
	Breeds		Season		
Natural/Artificial Number of Insemination	Charolais	Limousin	Cold (*n* = 110, %35.5)	Cool (*n* = 144, %46.4)	Hot (*n* = 56, %18.1)
1	13 (%17.11)	91 (%38.89)	42 (%38.18)	48 (%33.33)	14 (%25.00)
2	19 (%25.00)	61 (%26.07)	23 (%20.91)	48 (%33.33)	9 (%16.07)
3	13 (%17.11)	41 (%17.52)	18 (%16.36)	25 (%17.36)	11 (%19.64)
4	8 (%10.53)	19 (%8.12)	8 (%7.27)	7 (%4.86)	12 (%21.43)
5	8 (%10.53)	9 (%3.85)	7 (%6.36)	5 (%3.47)	5 (%8.93)
6	9 (%11.84)	7 (%2.99)	6 (%5.45)	7 (%4.86)	3 (%5.36)
7	6 (%7.89)	5 (%2.14)	6 (%5.45)	3 (%2.08)	2 (%3.57)
8	0 (%0)	1 (%0.43)	0 (%0)	1 (%0.69)	0 (%0)
^♣^Statistics	X^2^ = 27.5870 *P* < 0.0003		X^2^ = 27.1338 *P* < 0.0185		

^♣^Chi-square test was used to analyze the relationships presented in the table

**Table 5:** The relationship between sex and parturition ease, complications, parturition time and retained placenta rate

	Relationship			^♣^Statistics
^*^Sex	**Parturition ease**			
	**Easy**	**Difficult**	**Very Difficult**	
Female (%47.74)	100 (%67.57)	46 (%31.08)	2 (%1.35)	X^2^ = 25.8450 *P* < 0.0001
Male (%52.26)	64 (%39.51)	97 (%59.88)	1 (%0.62)	
	**Complications**			
	Uterine prolapse	Trauma	No	
Female	142 (%95.95)	3 (%2.03)	3 (%2.03)	X^2^ = 2.5922 *P* < 0.2736
Male	150 (%92.59)	9 (%5.56)	3 (%1.85)	
	**Parturition time**			
	Night parturitions	Day parturitions		
Female	64 (%43.24)	84 (%56.76)		X^2^ = 0.0001 *P* < 0.9953
Male	70 (%43.21)	92 (%56.79)		
	**Retanined placenta rate**			
	Expelled	Not expelled		
Female	143 (%96.62)	5 (%3.38)	Female (*n* = 148)	X^2^ = 2.4223 *P* < 0.1196
Male	150 (%92.59)	12 (%7.41)	Male (*n* = 162)	

^♣^Chi-square test was used to analyze the relationships presented in the table, ^*^ Female: n = 148, Male: n = 162

No relationship was identified between season and complications, parturition time, or retained placenta rate (Table 6). No significant differences in complications or parturition time were observed based on parturition ease. However, as parturition ease increased, the retained placenta rate decreased (X^2^ = 6.6971, *P* < 0.0351; Table 7). Uterine prolapse cases were more frequent during day parturitions. Complications were generally not observed during day parturitions (X^2^ = 6.2649, *P* < 0.0436), but complications were found to predispose to retained placenta (X^2^ = 12.4733, *P* < 0.0020; Table 8).

**Table 6:** The relationship between season and complications, parturition time, and retained placenta rate

	Relationship			^♣^Statistics
**Season**	**Complications**			
	**Uterine prolapse**	**Trauma**	**No**	
Cold (%42.26)	6 (%4.58)	5 (%3.82)	120 (%91.60)	X^2^ = 5.2392 *P* < 0.2636
Cool (%23.55)	3 (%4.11)	1 (%1.37)	69 (%94.52)	
Hot (%34.19)	3 (%2.83)	0 (%0)	103 (%97.17)	
	**Parturition time**			
	Night parturitions	Day parturitions		
Cold (%42.26)	56 (%42.75)	75 (%57.25)		X^2^ = 1.6759 *P* < 0.4326
Cool (%23.55)	36 (%49.32)	37 (%50.68)		
Hot (%34.19)	42 (%39.62)	64 (%60.38)		
	**Retanined placenta rate**			
	Expelled	Not expelled		
Cold (%42.26)	125 (%95.42)	6 (%4.58)		X^2^ = 3.1781 *P* < 0.2041
Cool (%23.55)	66 (%90.41)	7 (%9.59)		
Hot (%34.19)	102 (%96.23)	4 (%3.77)		

^♣^Chi-square test was used to analyze the relationships presented in the table, ^*^ Cold: n = 131, Cool/Warm: n = 73, Hot: n = 106

**Table 7:** The relationship between parturition ease and both complications and parturition time

	Relationship			^♣^Statistics
^*^ Parturition ease	Complications			
	Uterine prolapse	Trauma	No	
Easy (%52.90)	4 (%2.44)	2 (%1.22)	158 (%96.34)	X^2^ = 3.3157 *P* < 0.5065
Difficult (%46.13)	8 (%5.59)	4 (%2.80)	131 (%91.61)	
Very difficult (%0.97)	0 (%0)	0 (%0)	3 (%100)	
	Parturition time			
	Night parturitions	Day parturitions		
Easy (%52.90)	72 (%43.90)	92 (%56.10)		X^2^ = 0.7959 *P* < 0.6717
Difficult (%46.13)	60 (%41.96)	83 (%58.04)		
Very difficult (%0.97)	2 (%66.67)	1 (%33.33)		
	**Retanined placenta rate**			
	Yes	No		
Easy (%52.90)	160 (%97.56)	4 (%2.44)		X^2^ = 6.6971 *P* < 0.0351
Difficult (%46.13)	130 (%90.91)	13 (%9.09)		
Very difficult (%0.97)	3 (%100)	0 (%0)		

^♣^Chi-square test was used to analyze the relationships presented in the table, * Easy: *n* = 164, Difficult: *n* = 143, Very difficult: *n* = 3

**Table 8:** The relationship between complications and both parturition time and retained placenta rate

	Relationship		^♣^Statistics
^*^Complications	**Parturition time**		
	Night parturitions	Day parturitions	
Uterine prolapse (%3.87)	1 (%8.33)	11 (%91.67)	X^2^ = 6.2649 *P* < 0.0436
Trauma (%1.94)	3 (%50.00)	3 (%50.00)	
No (%94.19)	130 (%44.52)	162 (%55.48)	
	**Retanined placenta rate**		
	Yes	No	
Uterine prolapse (%3.87)	10 (%83.33)	2 (%16.67)	X^2^ = 12.4733 *P* < 0.0020
Trauma (%1.94)	4 (%66.67)	2 (%33.33)	
No (%94.19)	279 (%95.55)	13 (%4.45)	

^♣^Chi-square test was used to analyze the relationships presented in the table, ^*^Prolapsus Uteri: *n* = 12, Trauma: *n* = 6, No: *n* = 292

## Discussion

The breed factor is directly related to insemination success and calving difficulties, influencing labor costs, veterinary expenses, and calf losses, ultimately determining farm profitability. Reproduction, nutrition, and factors such as genetics are cornerstones of beef cattle breeding, supporting the ability to sustainably meet the growing global demand for red meat (Mwangi et al. 2019). The number of healthy weaned calves is crucial for the economic sustainability of beef cattle herds (Fernandez-Novo et al. 2020). Optimal reproductive performance in cattle requires strategic breeding practices, effective animal management, and a comprehensive understanding of the physiological traits specific to each breed. While lifetime productivity in meat breeds begins with the onset of puberty, herd productivity is shaped by key parameters such as age at first parturition, service period, number of inseminations required per pregnancy, and the number of calves successfully weaned (Diskin and Kenny 2014).

In this study, the fertility and parturition parameters of 9–11-month-old L and C heifers were compared. The higher insemination index observed in the C heifers likely reflects their later onset of puberty. Although the age of puberty varies in C heifers, one study reported that the age of first estrus in these breeds was 426 ± 42 days (Ménissier 1999); another study reported that this day was extended up to 623 days (Restle et al. 1999). By contrast, the average pubertal age for L heifers is approximately 470 days (Phocas et al. 2006). Despite initiating insemination at the same age in both breeds, the delayed puberty in C heifers, combined with their predisposition to accumulate excess fat before maturity, appears to have negatively affected their reproductive efficiency. Excessive fat accumulation, a recognized factor impairing fertility, likely exacerbated these effects (Hayırlı et al. 2019). Moreover, the higher feed efficiency in L heifers (Kayar and Inal 2019) may have enhanced their adaptability to environmental conditions compared to their C counterparts (Fidancı et al. 2022).

Differences in birth weight (BW) between breeds were pronounced, with C calves (46.1 ± 0.8 kg) exhibiting significantly higher BW compared to L calves (40.2 ± 0.3 kg). These findings are consistent with previous reports, which documented average BW values of 47.1 kg for C calves and 39.5 kg for L calves (Pelmuș et al. 2023). The higher BW observed in C calves is likely attributable to enhanced nutrient transfer during the final trimester, which may be associated with the breed’s inherently larger body frame. However, while increased BW in C calves may be advantageous in terms of growth potential, it can also elevate the risk of dystocia, potentially necessitating additional veterinary interventions and negatively affecting calf viability and overall farm profitability (Tohumcu and Tulan Tohumcu 2024). Furthermore, the lower BW observed in female calves and the positive association between extended gestation length and increased BW are consistent with patterns reported in other cattle breeds (Nelson et al. 2016; Coleman et al. 2021; Von Konigslow et al. 2022).

The influence of breed on the parturition process was apparent in this study. The dense muscle mass of C heifers may have contributed to a narrower birth canal, thereby increasing parturition difficulty (Zahradkova et al. 2010). Additionally, the greater feed conversion efficiency and BW of C calves compared to L calves likely heightened the challenges of parturition (Simčič et al. 2006; Bennett et al. 2021). The high incidence of dystocia in C heifers may also be tied to an inadequate age at conception (AC), as previous research suggests that an AC of 22–24 months reduces dystocia risks in dairy heifers (Coleman et al. 2022).

The economic implications of the number of inseminations required for pregnancy are significant (Cengiz et al. 2021). Studies indicate that approximately two inseminations per pregnancy are typical (Cengiz et al. 2021; Akbaş et al. 2022). The superior ability of L heifers to conceive within three inseminations may be attributed to optimal progesterone levels during insemination, which facilitate uterine readiness for implantation (Abay et al. 2021). By contrast, da Silva et al. (2020a, b) observed no effect of exogenous progesterone on pregnancy rates. The C breed is characterized by high weight gain and large body size. Feed restriction during the reproduction process may lead to lower performance compared to other breeds (Vaz et al. 2022). The higher pregnancy rates in C heifers through natural estrus could be influenced by elevated estrogen levels (Martinez et al. 2007) or disparities in live weight affecting dietary intake (NASEM  2021).

Research into the relationship between calf sex and parturition ease highlights consistent patterns. Studies have demonstrated that female calves are delivered with greater ease compared to males, a trend that was corroborated by our findings (Atashi et al. 2012; Gonçalves et al. 2019). However, our results revealed no significant association between calf sex and complications or retained placenta, indicating that post-parturition risks are independent of calf sex (Rezende et al. 2020). While some studies suggest male calves may increase the risk of retained placenta (Rohmah et al. 2023; Pajohande et al. 2023), others have proposed that calf sex may influence parturition timing (Üceş and Karakök 2007; Hess et al. 2016), a hypothesis supported by our findings.

The seasonal patterns of parturition timing observed in this study are consistent with established literature (Üceş and Karakök 2007). However, calf sex appears unaffected by seasonal factors, a trend that aligns with broader observations. The absence of seasonal effects on complications, parturition timing, and retained placenta rates may reflect the mild temperature ranges at the study location (< −10 °C or >  + 25 °C), as noted in climate analyses of the farm’s region (Köse and Sacakli 2021). Although direct temperature measurements were not taken, the interpretation is grounded in regional climate data.

Our findings also highlight the relationship between parturition ease and retained placenta rates. As parturition ease improved, retained placenta rates decreased, underscoring the role of dystocia in elevating retention risks. These results are consistent with investigations across various breeds (Gonçalves et al. 2019; Nogalski et al. 2024). The increased prevalence of uterine prolapse during daytime parturitions may stem from more frequent interventions and the use of forced calf extraction during daylight hours (Abdisa 2018). Furthermore, negative energy balance, micronutrient deficiencies, and complications have long been identified as contributing factors to retained placenta (Xiao et al. 2021). The complications documented in this study similarly exacerbated retained placenta rates, aligning with previous reports (Islam et al. 2012; Simoes et al. 2021).

## Conclusions

The presented study offers valuable insights for breeders evaluating C and L heifers in terms of fertility and parturition performance. The higher insemination index observed in C heifers indicates that achieving pregnancy in this breed presents greater challenges. In contrast, L heifers demonstrated significant advantages in both insemination efficiency and ease of parturition. This difference provides a notable benefit by reducing labor demands and the need for veterinary interventions. Consequently, L heifers emerge as a more suitable option for beef farms aiming to optimize fertility and minimize parturition complications due to their superior reproductive efficiency and ease of calving. These findings indicate that Limousin heifers may be more suitable for beef production due to their superior reproductive efficiency and lower incidence of dystocia, which can contribute to improved herd productivity and greater economic returns. However, this study was limited to two beef cattle breeds raised under standardized management and environmental conditions on a single farm. Therefore, the generalizability of the results to herds managed under different systems or environmental contexts may be restricted. Future studies should aim to include a broader range of breeds, larger and more diverse populations, and varying management conditions. Additionally, the incorporation of hormonal, metabolic, and genomic analyses would provide deeper insights into the physiological mechanisms underlying reproductive performance differences among breeds.

## Data Availability

The datasets generated during and/or analyzed during the current study are available from the corresponding author on reasonable request.
